# Identification of Cancer Stem Cell Molecular Markers and Effects of hsa-miR-21-3p on Stemness in Esophageal Squamous Cell Carcinoma

**DOI:** 10.3390/cancers11040518

**Published:** 2019-04-11

**Authors:** Zhikui Gao, Hui Liu, Yajuan Shi, Lihong Yin, Yong Zhu, Ran Liu

**Affiliations:** 1 Key Laboratory of Environmental Medicine Engineering, Ministry of Education, School of Public Health, Southeast University, Nanjing 210000, China; gaozhikuijy@163.com (Z.G.); syj799@163.com (Y.S.); lhyin@seu.edu.cn (L.Y.); 2 Chengdu Center for Disease Control and Prevention, Chengdu 610000, China; lh_kitty@163.com; 3 Department of Environmental Health Sciences, Yale School of Public Health, New Haven, CT 06510, USA; yong.zhu@yale.edu

**Keywords:** esophageal squamous cell carcinoma, cancer stem cell, Hsa-miR-21-3p, TRAF4

## Abstract

Cancer stem cells (CSCs) are closely related to tumor resistance and tumor recurrence in esophageal squamous cell carcinoma (ESCC). The lack of specific biomarkers to identify and isolate CSCs has led to the slow progression of research on CSCs in ESCC. Here, we established a method to identify and isolate CSCs in ESCC using fluorescence-activated cell sorting with combined surface biomarkers including CD71, CD271, and CD338. CD71^−^/CD271^+^/CD338^+^ subpopulation cells possessed more stem cell properties in proliferation, self-renewal, differentiation, metastasis, drug resistance, and tumorigenesis. We further explored possible roles that microRNAs played in stem cells. Using microarrays, we identified that has-miR-21-3p was highly expressed in positive sorted cells, and further functional and Luciferase reporter assays verified that has-miR-21-3p promoted proliferation and anti-apoptosis by regulating TRAF4. We further analyzed the relationship between hsa-miR-21-3p and ESCC in 137 patients with ESCC. Statistical analysis showed that up-regulation of hsa-miR-21-3p was associated with a high risk of ESCC. Collectively, we identified surface biomarkers of stem cells in esophageal squamous cell carcinoma, and discovered thathsa-miR-21-3p may be involved in stemness maintenance by regulating TRAF4.

## 1. Introduction

Esophageal cancer is the eighth most common cancer and ranks as the fourth highest cause of cancer-related mortality worldwide, with 456,000 new cases and 400,000 deaths in 2012 [1]. Esophageal squamous cell carcinoma (ESCC) is the principal histological type of esophageal cancer, with high incidence and mortality in China, Korea, Japan, and Africa. ESCC patients are more likely to be diagnosed at a later stage because there is a lack of specific biomarkers for diagnosis. The 5-year survival rate of esophageal cancer patients is no more than 20% [2]. The poor overall survival of patients with ESCC is mainly responsible for therapy resistance and recurrence. Finding novel, sensitive, and specific biomarkers for early diagnosis and elimination of therapy resistance has great potential to improve the outcomes of ESCC patients.

Cancer stem cells (CSCs) are considered to be closely related to the origin of cancer. Principle research for CSCs proposed that tumors are composed of a few CSCs, which have a strong capacity to self-renew, and a large proportion of common tumor cells that are derived from CSCs [3,4,5]. CSCs are believed to originate from stem cells with high tumorigenic properties. A large number of studies and clinical trials indicated CSCs are responsible for recurrence of ESCC [3,6]. Unfortunately, CSCs are not sensitive to chemotherapy and radiotherapy because abnormal modulating signaling pathways exist, such as Notch, Wnt, and Hedgehog [7,8,9]. Therefore, the study of CSCs in ESCC will provide a new window for ESCC research.

Because there is a lack of specific methods for identifying and isolating CSCs, research on CSCs in ESCC has progressed slowly. Currently, CSCs in ESCC are mainly isolated with flow cytometry using multiple protein markers and fluorescent probes such as CD44, Side-population (SP), CD271, Aldehyde dehydrogenase (ALDH), and CD90 [10,11,12,13]. However, these biomarkers are not specific for ESCC, which have been reported to be expressed in other tumor CSCs; there are no unique biomarkers for CSCs in ESCC. Even the expression of these biomarkers varies considerably in different kinds of CSCs. A combination of multiple biomarkers can greatly improve the specificity for stem cell sorting. In this study, various combinations of CD71, CD338, CD271, and CD49f have been considered and tested in ECa9706, ECa109, KYSE50, and CAES17. Finally, CD71^−^/CD271^+^/CD338^+^ were verified to be positive biomarkers for identifying CSCs in ESCC.

The exact mechanisms for CSCs involved in ESCC tumor genesis remain largely unknown. MicroRNAs (miRNAs) are important regulators in CSCs by binding to the 3′UTR region of target-mRNAs. miRNA networks in CSCs play an important role in the maintenance of stemness, which was considered to be a potential target in ESCC therapy [14,15,16,17]. In this study, we discovered the expression of hsa-miR-21-3p is up-regulated in CSCs of ESCC, and hsa-miR-21-3p can promote cell proliferation but suppress cell apoptosis. We further identified TRAF4 as the direct target of hsa-miR-21-3p.

## 2. Results

### 2.1. CD71, CD27,1 andCD338 Are Potential Biomarkers for Cancer Stem Cell (CSC) Sorting in Esophageal Squamous Cell Carcinoma (ESCC)

**Figure 1:**
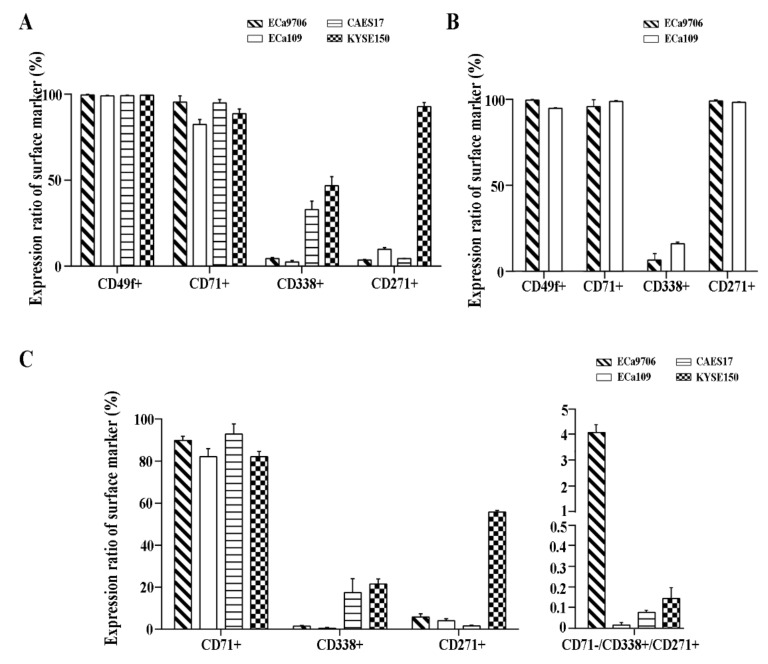
Expression of CD49f, CD71^−^, CD271^+^, and CD338^+^ in esophageal squamous cell carcinoma (ESCC). (**A**) Expression ratios of surface markers in different cell lines. (**B**) Expression ratios of surface markers in cell lines stained with the four surface markers together. (**C**) Expression ratios of CD71, CD271, and CD338 in different cell lines.

Through a literature review, four candidate stem surface markers (CD49f, CD71, CD338, and CD271) were selected for CSC sorting in ECa9706, ECa109, CAES17, and KYSE150. We firstly detected CD49f, CD71, CD338, and CD271 when stained individually; CD49f and CD71 were detected with high positive rates, and CD338 and CD271 were expressed with low positive rates (Figure 1A). When labeled together (Figure 1B), PE-CY5 (CD49f) and APC (CD271) interfered with each other. Considering that the positive rate of CD49f was almost 100% in four cell lines, which was less meaningful for cell sorting, we selected CD271 and CD338 as positive and CD71 as negative surface biomarkers. In multiple fluorescently labelled cells, the detectable rate of CD71, CD271, and CD338 coincided with single-labeled cells. The percentage for the CD71^−^/CD271^+^/CD338^+^ subpopulation ranged from 0.02% to 4.12% in four cell lines (Figure 1C).

### 2.2. CD71^−^/CD271^+^/CD338^+^ Subpopulation Cells Possessed More Stem Cell Properties

**Figure 2:**
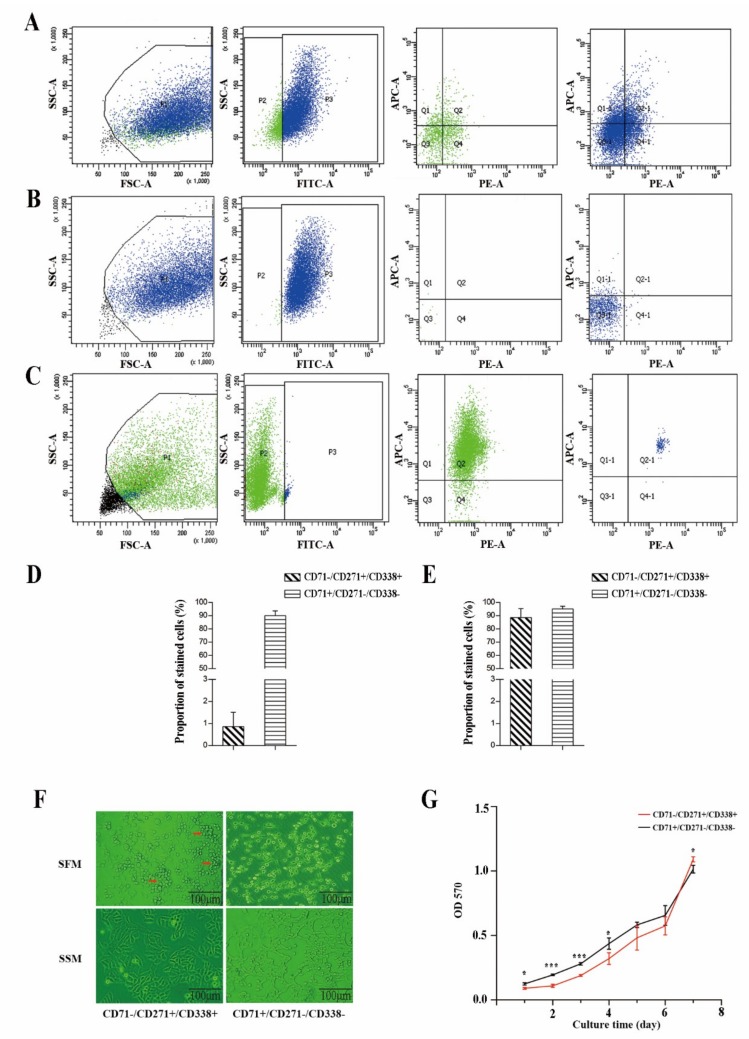
Combination of CD71^−^, CD271^+^, and CD338^+^ can be used for isolating cancer stem cells (CSCs) in ESCC. (**A**) Expression of CD71, CD271, and CD338 in Eca9706 in multiple stained cells. (**B**) Expression of surface markers in CD71^−^/CD271^+^/CD338^+^ cells just after sorting. (**C**) Expression of surface markers in CD71^+^/CD271^−^/CD338^−^ cells just after sorting. (**D**) Positive rate of CD71^−^/CD271^+^/CD338^+^ and CD71^+^/CD271^−^/CD338^−^ cells in Eca9706 for sorting. (**E**) Recovery rate of sorted CD71^−^/CD271^+^/CD338^+^ and CD71^+^/CD271^−^/CD338^−^ cells. (**F**) Growth status of the two subpopulations of cells in serum-free-DMEM-F12 medium (SFM) and serum-supplied medium (SSM). (**G**) Growth curve of the two subpopulations of cells in SSM. *t*-Tests (independent samples) were used to compare the results between two groups: * *p* < 0.05, *** *p* < 0.001.

In EC9706, the rates for positive (CD71^−^/CD271^+^/CD338^+^) and negative (CD71^+^/CD271^−^/CD338^−^) subpopulations were 0.86% and 90.0%, respectively. The recovery purities for positive and negative subpopulations were 88.6% and 95.1%, respectively (Figure 2A–E).

We then cultured sorted cells using serum-supplied medium with 10% fetal bovine serum (SSM) and serum-free-DMEM-F12 medium (SFM), respectively. In SSM, positive cells formed into cell spheres, but the negative cells were dispersed. In SFM, cells grew into slices. No significant differences in morphology between the two subpopulations were observed (Figure 2F).

The growth curve was measured using an Thiazolyl blue tetrazolium bromide (MTT) assay. Sorted cells were cultured in SFM. In first four days the negative subpopulation grew faster than the positive, but from day four to day six the difference in growth disappeared. By day seven the growth rate of the positive subpopulation exceeded the negative (Figure 2G).

#### 2.2.1. Proliferative Capacity

**Figure 3:**
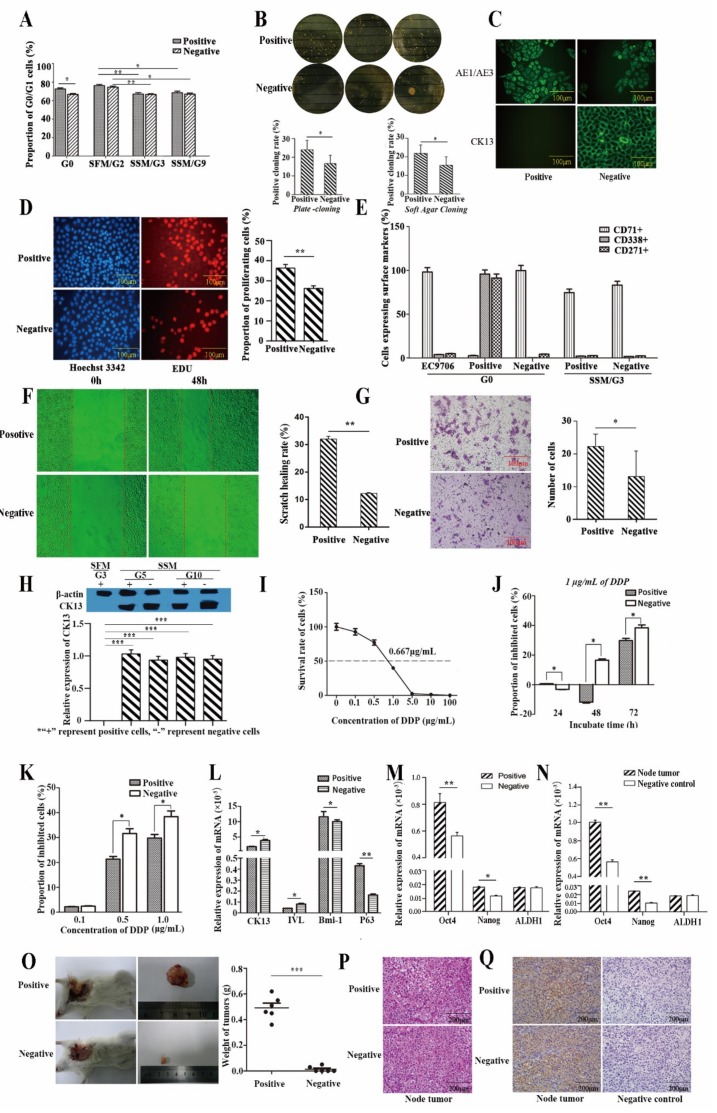
CD71^−^/CD271^+^/CD338^+^ subpopulations of cells possessed more stem cell properties. (**A**) Cell cycle analysis of the two subpopulations of cells using flow cytometry. (**B**) Self-renewal ability was detected by plate-cloning and soft agar-cloning experiments. (**C**) Immunofluorescence analysis of Cytokeratin AE1/AE3 and CK13 in two subpopulations of cells when cultured for three generations. (**D**) Proliferation of two subpopulations of cells when cultured in SSM and SFM. (**E**) Expression of CD271, CD71, and CD338 in different subpopulations of cells. (**F**) Migration ability of two subpopulations of cells detected by scratch-healing experiments. (**G**) Result of invasiveness detected by a Transwell assay. (**H**) The expression of CK13 detected by Western blot. (**I**) Half maximal inhibitory concentration (IC50) of cisplatin (DDP) for positive subpopulation cells. (**J**) Inhibitory effect of 1µg/mL DDP on two subpopulations of cells at different times. (**K**) Inhibitory effects of different drug concentrations on two subpopulations of cells after 120 h. (**L**,**M**) Expression of mRNAs related to stemness in sorted cells. (**N**) Expression of mRNAs related to stemness in tissues. (**O**) Transplantation of two subpopulations of cells in NOD/SCID mice. (**P**) Pathological analysis of the transplanted tumors using staining techniques. (**Q**) Immunohistochemical analysis of AE1/AE3 in node tumors and negative control. *t*-Tests (independent samples) were used to compare the results between two groups: *, *p* < 0.05; **, *p* < 0.01; and ***, *p* < 0.001.

We detected the cell cycle of cells cultured in SSM and SFM. For the positive subpopulation, the proportion of G0 cells was significantly higher than the negative just after sorting. As time went on, the difference between the two subpopulations faded away when cultured in SSM (Figure 3A). Coincidentally, the proliferate rate for the positive subpopulation was significantly higher than the negative (36.33% vs. 26.18%) (Figure 3D).

#### 2.2.2. Self-Renewal Ability

A plate clone formation assay showed that the positive subpopulation had a higher colony formation rate than the negative (24.00% ± 2.08% vs. 16.63% ± 1.42%, *p* < 0.05). In addition, in the soft agar assay the positive subpopulation also had a higher colony formation rate than the negative (21.93% ± 4.50% vs. 15.53% ± 4.51%, *p* < 0.05) (Figure 3B).

#### 2.2.3. Differentiative Capacity

For the positive subpopulation, when cultured in SSM, the expression of surface markers representing differentiation (CD71) increased, while the expression of surface markers representing stemness (CD271 and CD338) decreased. As time went on, the expression of CD271, CD71, and CD338 became similar to negative and non-sorting cells (Figure 3E).

As an important cytokeratin, cytokeratin 13 (CK13) reflects the differentiation of epithelial cells [18]. Immunofluorescence analysis showed that Cytokeratin AE1/AE3 and CK13 were mainly expressed in the cell membrane (Figure 3C). Then, the expression of CK13 was analyzed by Western blot. No CK13 was expressed in positive subpopulation cells when cultured in SFM, and there was no difference in expression of CK13 between the two subpopulations of cells when cultured in SSM (Figure 3H).

#### 2.2.4. Metastasis Ability

A scratch wound healing assay (Figure 3F) and a Transwell chamber in vitro invasion assay (Figure 3G) showed that the positive subpopulation was more aggressive and migratory than the negative.

#### 2.2.5. Drug Resistance

As a common chemotherapeutic agent for ESCC, cisplatin (DDP) was selected for drug resistance research [19]. The IC50 (0.667 µg/mL) of DDP for EC9706 was determined by the improved Karber’s method (Figure 3I). We detected growth inhibition in SSM with 1 µg/mL of DDP. Interestingly, cell growth was initially promoted, but as time went on, growth-promotion changed to growth-inhibition and the inhibitory effect of DDP on the negative subpopulation cells was more significant (Figure 3J). When cultured with 0.1 µg/mL, 0.5 µg/mL, and 1.0 µg/mL DDP for 120 h, both positive and negative cells were inhibited, and the inhibitory effect gradually increased with increasing concentrations, where the negative subpopulation cells were more sensitive to DPP treatment (Figure 3K).

#### 2.2.6. Gene Expression Related to Stemness 

The key transcriptional factors for epithelial cells (P63, Bmi-1), differentiation markers for epithelium (Involcrin, CK13), and stemness (Oct4, Nanog and ALDH1) [18,20,21,22,23,24,25] were detected by RT-QPCR. P63, Bmi-1, Oct4, and Nanog were expressed higher in the positive subpopulation, while CK13 and Involcrin were expressed significantly higher in the negative subpopulation. (Figure 3L–N).

#### 2.2.7. Tumor Xenograft in Nude Mice

The positive subpopulation was strongly tumorigenic, with a 100% tumor formation rate in six NOD/ Severe Combiae DificiEncy (NOD/SCID) mice, where a tumor formation rate of only 33.33% was seen in the negative. The tumor weights of the positive subpopulation ranged from 0.36 g to 0.62 g, which were significantly higher than the negative (range from 0.03 g to 0.05 g) (Figure 3O). Cells in the tumors showed obviously malignancy. Hematoxylin-eosin (HE) staining showed the tumor cells were patchy or striate, they had spindle or polygonal shapes, and the cytoplasm of the cells were light-stained while the nuclei were dark-stained (Figure 3P). Immunocytochemistry of human AE1/AE3 confirmed tumor cells were derived from human lines (Figure 3Q). RT-QPCR showed Oct4 and Nanog were expressed higher in the node tumor derived from the positive subpopulation.

### 2.3. Differently Expressed miRNAs Are Vital for Cell Stemness

**Figure 4:**
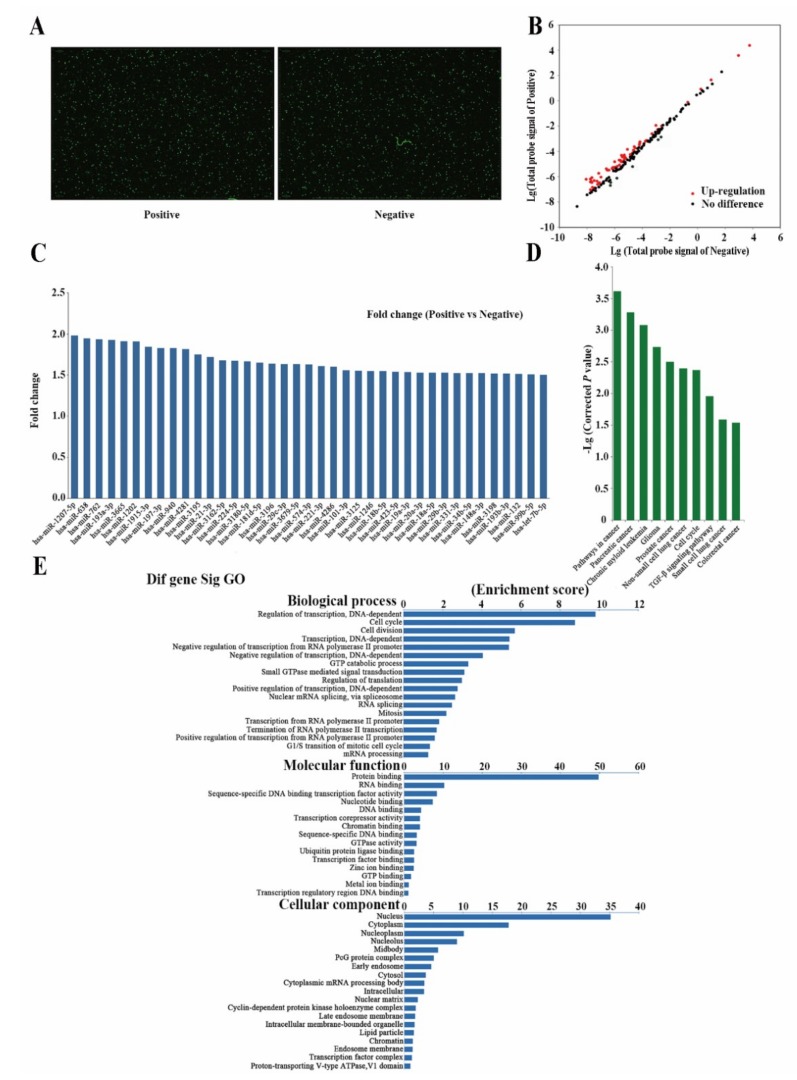
Analysis of differently expressed miRNAs between two subpopulations of cells. (**A**) Expression map of microarray hybridization fluorescence. (**B**) Scatterplot of expression of miRNAs detected by microarray. (**C**) Differently expressed miRNAs between the two subpopulations of cells. Detailed data are listed in Table S1. (**D**) Gene ontology (GO) analysis of the selected differently expressed miRNAs. The selected miRNAs included hsa-let-7b, hsa-miR-18a, hsa-miR-18b, hsa-miR-19a, hsa-miR-20a-3p, hsa-miR-21-3p, hsa-miR-29b, hsa-miR-29c, hsa-miR-34b-5p, and hsa-miR-99b. (**E**) Common pathway analysis related to stemness.

Different expressions of miRNAs in the sorted cells were detected using an miRNA chip (Figure 4A,B). Thirty-nine up-regulated miRNAs, including hsa-miR-18a-5p, hsa-miR-18b-5p, hsa-miR-29b-3p, hsa-miR-29c-3p, and hsa-let-7b were identified (Figure 4C). Gene ontology (GO) analysis revealed that these miRNAs were mostly enriched in transcriptional regulation, cell cycle, and cell division. In biological processes (in terms of molecular function), protein binding, RNA binding, and sequence-specific DNA binding were enriched. In the cellular component, these miRNAs were mostly enriched in the nucleus, cytoplasm, and nucleoplasm (Figure 4D). Pathway analysis revealed these miRNAs were mostly involved enriched in cell cycle regulation pathway and TGF-beta signaling pathway (Figure 4E).

### 2.4. Analysis of Differently Expressed mRNAs between Two Subpopulations of Cells

**Figure 5:**
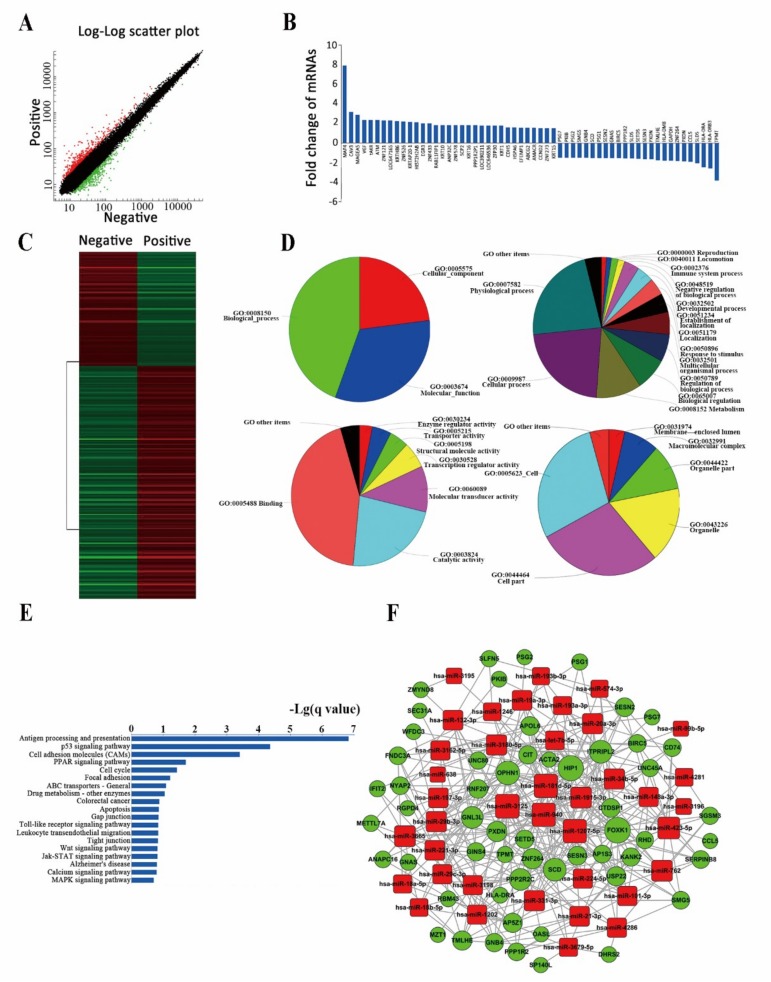
Analysis of differently expressed mRNAs between two subpopulations of cells. (**A**) Scatter plot of differently expressed mRNAs detected by microarray. (**B**) Partial differently expressed mRNAs. Detailed data are listed in Table S2. (**C**) Distinct expressions between positive and negative subpopulations of cells revealed by clustering analysis. (**D**) GO analysis of the selected differently expressed mRNAs. (**E**) Pathway analysis for differently expressed mRNAs in the two cell subpopulations. (**F**) Predicted miRNA-associated mRNA network participates in maintenance of cell stemness.

Using a genome microarray, 303 differently expressed mRNAs were detected between two subpopulations: 205 were up-regulated (including MAP4 and ATM) and 98 were down-regulated in the positive subpopulation (including PSG7, PKIB, and SMG5) (cut-off = 1.5) (Figure 5A–C).

GO analyses of biological processes revealed these mRNAs were mostly enriched in physiological processes, cellular processes, and metabolic regulation. GO analyses of molecular functioning revealed transcription regulation, epidermal development, and signal transduction were closely related with these mRNAs. For cellular components, binding, catalysis, and molecular transduction were mostly enriched (Figure 5D). Pathway analysis revealed these differently expressed mRNAs were closely related with Antigen processing and presentation, p53 signaling pathway, cell adhesion molecules and PPAR Signaling pathway (Figure 5E).

We further predicted the miRNA-associated mRNA network as it participates in maintenance of cell stemness according to the expression profiles of miRNA and mRNA (Figure 5F).

### 2.5. Hsa-miR-21-3p Is Critical for the Stemness Maintenance of CSCs

**Figure 6:**
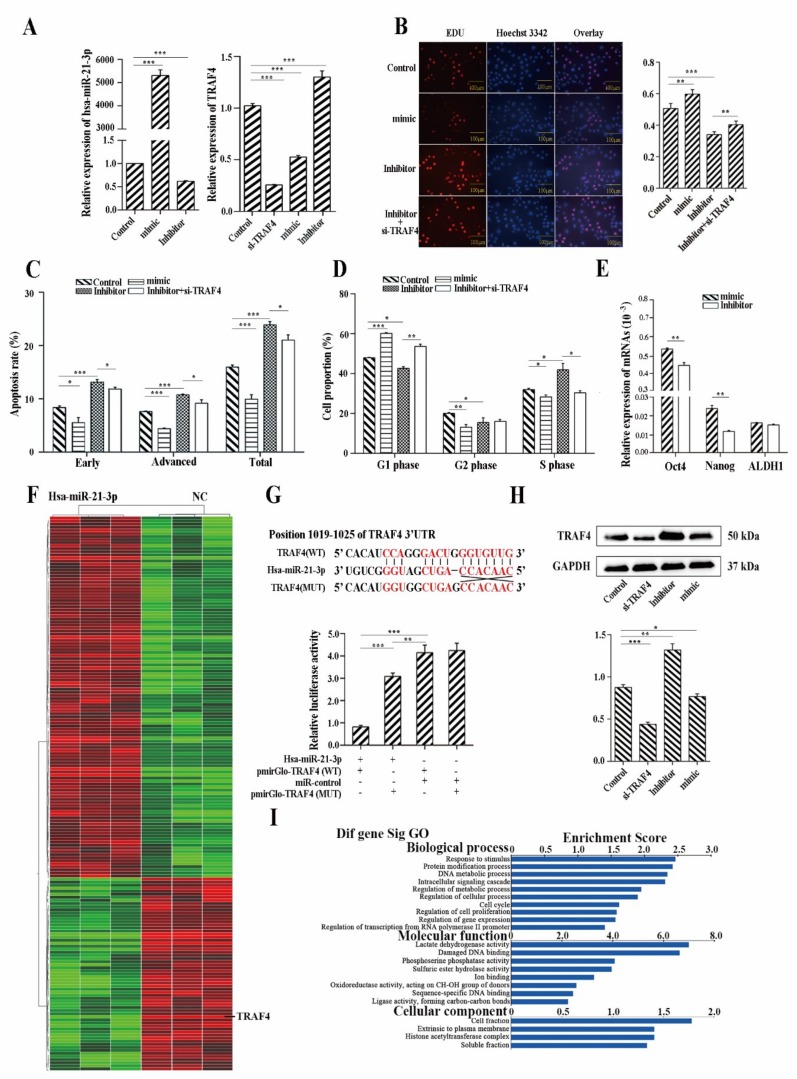
Hsa-miR-21-3p is critical for stemness maintenance of CSCs via inhibition of TRAF4. (**A**) Relative expression of hsa-miR-21-3p and TRAF4 detected by RT-QPCR. (**B**) Result of cell proliferation detected by EdU. Scale Bar: 100 μm. (**C**) Result of apoptosis detected by flow cytometry. (**D**) Result of cell cycle. (**E**) Expression of stemness-related genes. (**F**) Cluster analysis based on expression levels of mRNAs between cells over-expressing hsa-miR-21-3p and controls. (**G**) Dual-Luciferase Reporter gene assay showed TRAF4 mRNA is a direct target of miR-21-3p. (**H**) Western blot analysis of TRAF4. (**I**) GO analysis of differently expressed mRNAs. Detailed data are listed in Table S3. *t*-Tests (independent samples) were used to compare the result between two groups: *, *p* < 0.05; **, *p* < 0.01; and ***, *p* < 0.001.

We constructed cell lines over-expressing and under-expressing hsa-miR-21-3p using an hsa-miR-21-3p mimic and an inhibitor (Figure 6A). 5-ethynyl-2’-deoxyuridine (EdU) apoptosis assay and cell cycle assay demonstrated that hsa-miR-21-3p promoted cell proliferation, migration, and invasion in ECa9706, while it decreased cell apoptosis (Figure 6B–D). RT-QPCR showed hsa-miR-21-3p could significantly promote expression of Oct4 and Nanog (Figure 6E).

Differently expressed mRNAs between the hsa-miR-21-3p overexpressed cell line and control were detected using a genome-wide mRNA microarray. Sixty-three down-regulated and 118 up-regulated mRNAs were detected in ECa9706 that overexpressed hsa-miR-21-3p (Figure 6F).

Results of the GO analysis are shown in Figure 6I. hsa-miR-21-3p was closely related to protein modification, cell proliferation, cell cycle phase, and the DNA metabolic process. In molecular function terms, DNA and ion binding, activity of lactate dehydrogenase, ligase activity, and phosphoserine phosphatase were critical functions influenced by hsa-miR-21-3p. Pathway enrichment analysis based on the Kyoto Encyclopedia of Genes and Genomes (KEGG) database showed the differentially expressed genes were involved in a total of six pathways (*p* < 0.01), which were critically relevant to tumor proliferation and evading apoptosis, such as Notch, MAPK, and Wnt signaling pathway.

To investigate the underlying mechanisms of miR-21-3p-induced cell function, we searched for the possible target genes of miR-21-3p using two reduction algorithms: miRanda and miRWalk. Several genes, including TRAF4, which have been reported to be corrected with cell proliferation and apoptosis, were selected for further analysis. Simultaneously, TRAF4, a tumor necrosis factor receptor-associated factor, was significantly down-regulated in expression profiling, as referenced before. 

RT-QPCR and Western blotting showed that the over-expression of hsa-miR-21-3p significantly down-regulated both mRNA and protein expression of TRAF4. Conversely, down-regulation of hsa-miR-21-3p significantly up-regulated the expression of TRAF4 (Figure 6A,H). To validate whether TRAF4 was the direct target gene of miR-21-3p, TRAF4 3′UTR sequences were cloned into a dual-luciferase reporter plasmid for the Dual-Luciferase Reporter assay. We then constructed plasmids that contained mutated possible binding sites. The relative luciferase activity was significantly reduced (nearly 80.0%) by hsa-miR-21-3p when the reporter plasmid carried the wild type TRAF4 3′UTR, but no significant suppression was observed in the negative control plasmid; for cells transfected with mutant plasmid, only 25.6% of the relative luciferase activity was reduced (Figure 6G), which suggested miR-21-3p bound directly to the predicted TRAF4 3′UTR and negatively regulated TRAF4 expression. We further confirmed that down-regulation of TRAF4 could partly rescue the functional changes in proliferation, cell cycle, and apoptosis in EC9706 (Figure 6B–D).

### 2.6. Hsa-miR-21-3p May Be a Potential Biomarker for Early Diagnosis of ESCC

We collected 137 cases of ESCC patients confirmed by pathology in the department of thoracic surgery. ESCC tissues and their corresponding adjacent normal tissues were collected for QRT-PCR. Hsa-miR-21-3p was significantly up-regulated in cancer tissues (Table 1). We further analyzed the relationship between the expression of hsa-miR-21-3p and the risk for esophageal cancer using logistic regression analysis. The risk for ESCC increased when expression of hsa-miR-21-3p was up-regulated (Table 2), suggesting hsa-miR-21-3p may play an oncogenic role in ESCC.

## 3. Discussion

CSCs were mainly isolated using fluorescence-activated cell sorting (FACS) or immune-magnetic beads. (Antibody-mediated cell sorting using FACS is more suitable and straightforward to purify rare populations of CSCs in tumors.) Different panels of biomolecules were identified to detect and isolate these CSCs in various cancers. However, research progress on CSC sorting in ESCC has been hampered by the lack of suitable biomarkers for prospective isolation. It has been reported that CD44, CD133, ALDH (Aldehyde dehydrogenase), CD271, CD90, and Side-population (Hoechst 33,342 dye exclusion) are potential biomarkers used to identify cancer stem cells in ESCC. Still, there are lots of challenges and limitations. Usually these biomarkers have low specificity for rendered populations, and many biomarkers have no direct evidence in demonstrating the stemness. Multiple biomarkers could improve the specificity of cell sorting, and purer CSCs could be obtained with a combined selection rather than a single selection. 

In this study we identified three potential biomarkers for CSC sorting including CD271, CD338, and CD71. CD271 is also called p75 neurotmphin receptor (p75NTR), and in ESCC, cells expressing CD271 were reported to have higher self-amplifying and self-renewal capacities than cells not expressing CD271 [26]. The expression of CD271 was reported to be closely related with the survival and maintenance of cancer. In addition, the expression of stem cell-associated genes was dependent on CD271 [27]. Kojima discovered that CD271-positive cells have enhanced CSC properties that are mitotically quiescent in ESCC. CD338, also called ABCG2, is an isoform of an ATP-binding cassette transporter. The overexpression of ABCG2 was reported to be correlated with lymph node metastasis in ESCC patients. ABCG2 is considered to be a potential biomarker for CSCs in ESCC, and ABCG2-positive cancer seemed to produce more stemness [28]. CD71 is also known as transferrin receptor protein 1(TRf1), and CD71 has been used as a surface biomarker to isolate hemopoietic stem cells. In ESCC, CD71 is reported to be correlated with tumorigenic properties. The combination of CD71^−^/CD338^+^/CD271^+^ can be satisfactorily used to isolate CSCs in ESCC with a high specificity and efficiency, which can provide us with new strategies for further research on CSCs in ESCC.

It is widely believed that miRNAs are critical during stem cell epigenesis. It has been shown that the expression patterns of miRNAs changes during the differentiation of embryonic stem cells, which suggests miRNAs may play important roles in maintaining the pluripotency and self-renewal capacities of ES cells, and these miRNAs may also serve as molecular markers for ES cells. In addition, miRNAs played a key role in the process of proliferation and differentiation of hematopoietic cells, fats, nerves, muscles, and cardiomyocytes. Similarly, in CSCs, miRNA expression profiles were different from normal cancer cells. miR-135a can inhibit the development of Cancer Stem Cell-Driven Medulloblastoma by repressing Arhgef6 Expression [29]. Peng et al. [17] demonstrated that the miRNA-103/107 family can promote stem cell phenotypes by targeting ribosomal kinase p90RSK2. Liu et al. [30] discovered that miRNA-148b suppressed CSCs by targeting neuropilin-1 in hepatocellular carcinoma. However, there are few studies on the expression of miRNA in CSCs of ESCC. In this study, we detected miRNA and mRNA expression profiles in positive and negative cell subpopulations of CSCs. Fifty-four differently expressed miRNAs and 303 differently expressed mRNAs were discovered. Biological analyses revealed that differential expression of miRNAs and mRNAs are involved in transcriptional regulation, cell cycle regulation, cell differentiation regulation, and RNA splicing, which are closely with the maintenance of CSCs. miRNAs are potentially critical for the maintenance of CSCs in ESCC.

It has been reported that hsa-miR-21-3p can inhibit proliferation and invasion in ovarian cancer cells. In hepatocellular carcinoma, hsa-miR-21-3p inhibited tumor cell growth and promoted apoptosis [31,32,33]. However, in colorectal cancer hsa-miR-21-3p was upregulated and promoted cell migration and invasion [34], which revealed hsa-miR-21-3p played different roles in different tumors. In bone marrow mesenchymal stem cells, hsa-miR-21-3p had an abnormal expression [35]. It seems that hsa-miR-21-3p is potentially related with the maintenance of stemness. We detected that hsa-miR-21-3p was differently expressed between two subpopulations of cells. Then, we demonstrated hsa-miR-21-3p could promote cell proliferation, migration, and invasion in ESCC. We further identified TRAF4 as a direct target of has-miR-21-3p using a Dual-Luciferase Reporter assay and Western blot assay. TRAF4, as a strong evolutionary conservation gene, reinforced the idea that it exerted important biological functions [35]. The subcellular localization of TRAF4 has been controversial for years. Indeed, TRAF4 has been detected at the cell membrane, in the cytoplasm, and in the nucleus [36]. Several reports discovered that TRAF4 might be a regulated gene of p53 (mediating cell cycle arrest), DNA repair, and apoptosis of cells, and it was associated with the ability of responding to cellular stress [37], colony formation [38], and squamous cell carcinoma of the head and neck differentiation [39]. Kedlinger et al. [40] implicated TRAF4, in one of the emerging TJ-dependent signaling pathways, responded to cell polarity by regulating the cell proliferation/differentiation balance and, subsequently, epithelium homeostasis in TRAF4-deficient mice and drosophila. Research also suggested that TRAF4, as a mediator in the TNF-induced signaling pathway leading to activation of p70S6K, inhibited Fas-induced apoptosis [41]. Xin et al. [42] reported that TRAF4 can directly act on p75 NTR, and in this way the activation of NF-kB was inhibited. Interestingly, the expression of p75 NTR(CD271) was positive in our isolated stem cells. The hsa-miR-21-3p/TRAF4 axis potentially promoted cell proliferation by acting on p75 NTR. At the same time, by regulating signal transduction of NF-kB, cell apoptosis was inhibited. Our results supplied understanding of CSCs in ESCC, that the hsa-miR-21-3p/TRAF4 axis may potentially be a new target for inhibiting ESCC.

CSCs are considered to be closely related to tumor genesis and tumor recrudescence, which undergoes changes in early stages of tumor development. Differently expressed molecules may be used as biomarkers for early diagnosis and treatment. To detect the possibility of has-miR-21-3p as a biomarker for ESCC, we analyzed the relationship between the expression of has-miR-21-3p and the risk for ESCC in tissues collected during surgery. Our findings indicated miR-21-3p might serve as a biomarker for the diagnosis of ESCC.

## 4. Materials and Methods

### 4.1. Population

Esophageal carcinoma tissues and their corresponding normal non-tumor tissues (from adjacent 3 cm) were surgically collected between 2009 and 2010, and stored in tubes at −80 °C. A total of 137 cases of esophageal carcinoma patients, ranging from 43 to 80 years old, including 94 males and 43 females, were collected. All patients had not been treated with chemoradiation. Written informed consent was obtained from all subjects prior to recruitment to the study. Ethical approval was provided by the Institutional Review Board of the Southeast University-Affiliated Zhongda Hospital (Nanjing, China) (Approval no: 2011ZDL002.0, 24 February 2011).

### 4.2. Animal and Cell Lines

NOD/SCID mice were purchased from Beijing Vital River Laboratory Animal Technology Co., Ltd. (Beijing, China). The mice were female, aged 3 to 4 weeks, and weighed 21–25 g. The animals were housed and maintained in specific pathogen-free (SPF) shelves with a constant temperature (20–26 °C) and constant humidity (50–56%). Human ESCC cell lines ECa9706, ECa109, KYSE150, and CAES17 were provided by Key Laboratory of Environmental Medicine Engineering, Ministry of Education, School of Public Health, Southeast University. Cells not sorted were grown in RPMI-1640 containing 10% fetal bovine serum (Gibico, Grand Island, NY, USA), 100 U/mL penicillin–streptomycin solution (Gibico), and 200 mM L-glutamine (Invitrogen) at 37 °C in an incubator containing a 5% CO_2_ humidified atmosphere. The sorted cells were cultured in SSM and SFM with 10 ng/mL of EGF and bEGF (Invitrogen, Carlsbad, CA, USA). EC9706 cells over-expressing and under-expressing hsa-miR-21-3p were obtained using Micron™ miRNA mimic and inhibitor (RiboBio, Guangzhou, China). siRNAs used for decreasing TRAF4 were synthesized by RiboBio (forward, ATCCGAAAGCAGTGTGAACACTCCTTTCTTTCGTTAGGCTTGAATGAAGAACGAG; reverse, AGCAATAGTCGGTTCTGATTTCCAGTCTTACCAAAGCGTTAGGAACCGCGAAATTC). Lipofectamine^®^ RNAiMAX reagent (Thermo Fisher, Waltham, MA, USA) was used for transfection according to the manufacturer’s instructions.

### 4.3. Antibodies and Reagents

Anti-cytokeratin 13 antibody and anti-pan cytokeratin antibody (AE1 + AE3) were obtained from Abcam (Cambridgeshire, UK). GAPDH, β-actin, and TRAF4 antibodies were obtained from Cell Signaling Technology (CST, Danvers, MA, USA). The secondary anti-rabbit IgG1 HRP-conjugated and anti-mouse IgG HRP-conjugated antibodies were purchased from Abcam and CST, respectively.

### 4.4. Flow Cytometry and Cell Sorting

PE labeled anti-ABCG2 (CD338), FITC labeled anti-TfR1 (CD71), APC labeled anti-p75NTR (CD271), ALXA FLOUR^®^ 647 labeled anti-p75NTR (CD271), and PE-CY5 labeled anti-Integrinα6 (CD49f) were purchased from BD Biosciences (San Jose, CA, USA). Cells were marked according to the manual of regents. Live cells were counted using Trypan blue exclusion. Fluorescence was analyzed and sorted on a total of 1 × 10^4^ cells per sample using a flow cytometer (Facs Aria II; Becton Dickinson, Mountain View, CA, USA). The number of cells sorted depended on the requirements of the assay.

### 4.5. Apoptosis and Cell Cycle

Cell apoptosis was quantified using the Annexin V-FITC Apoptosis Detection Kit (KGA107, Keygen Biotech, Nanjing, China) according to the manufacture’s protocol. Flow cytometry (PI staining) was used to detect cell cycle using the PI cell cycle Detection Kit (KGA107, KeyGEN Biotech) according to the manufacture’s protocol.

### 4.6. Cell Proliferation Assay

Cells with a density of 1 × 10^4^ cells/well on 96-well plates were quantified using a 5-ethynyl-2′-deoxyuridine (EdU) labeling/detection kit (Ribobio, Guangzhou, China) to detect proliferation. Firstly, 50 mM EdU was applied to the cultures, and the cells were grown for an additional 2 h. Then, the cells were fixed with 4% formaldehyde in PBS for 30 min and incubated with glycine for 5 min. After washing with PBS and 0.5% TritonX-100 in PBS, the cells were incubated with 1× Apollo dye at room temperature in darkness for 30 min. Lastly, the cells were washed with 0.5% TritonX-100 in PBS and methanol, and they were incubated with 1× Hoechst 33342 dye at room temperature in darkness for 30 min. After labeling, cells were preserved with 100 μL PBS. Analyses of cell proliferation (ratio of EdU + to the total) were performed using images of five randomly selected fields obtained on a fluorescence microscope. Assays were performed in five parallels.

### 4.7. Plate Cloning Assay

The cells were suspended and cultured for sorting with SFM, and 1 × 10^2^ cells/well were planted on 96-well plates. After 14 d of culture, colony forming efficiency was calculated only for clones containing more than 50 cells.

### 4.8. Soft Agar Cloning Assay

Agaropectin was prepared, containing 0.6% and 0.2% of low melting point agar for the bottom and upper layers, respectively, using SFM. A total of 5 × 10^2^ cells/well were planted in 6-well plates. Clones were counted after 3 weeks under a microscope (Olympus, Tokyo, Japan).

### 4.9. Scratch-Healing Experiment

Sorted cells were planted in 6-well plates with SFM. When cells reached 90% confluence, the cells were scratched with a standard 10 µL pipette tip. Then, the plate was washed to remove cell debris, freshened with medium, and cultured for 48 h. After 48 h the size of wound was observed and measured under a microscope.

### 4.10. Invasive Experiment

Cell migration assays were performed using 8.0 μm Transwell chamber (Corning, Corelle, NY, USA). We first set a layer of Matrigel (Corning), and 5 × 10^4^ cells were seeded into the upper chamber in SFM, while the lower chamber was filled with DMEM-F12 containing 50% fetal bovine serum. Cells were then cultured for 24 h, colored, and the number of invasive cells were counted.

### 4.11. RNA Extraction and Genome-Wide mRNA Microarray

Total RNA was isolated by Trizol reagent (Invitrogen) using the standard method. The RNA samples were quantified with a Nanodrop spectrophotometer (Thermo). Human miRNA V16.0 (Agilent, Santa Clara, CA, USA) was used to detect miRNA profiles in positive (CD71^−^/CD271^+^/CD338^+^) and negative (CD71^+^/CD271^−^/CD338^−^) cells. A Roche NimbleGen expression chip was used to detect mRNA profiles in positive (CD71^−^/CD271^+^/CD338^+^) and negative (CD71^+^/CD271^−^/CD338^−^) cells. A HumanHT-12 v4 Expression Bead Chip Kit (Illumina, Santiago, CA, USA) was used to detect samples transfected with miR-21-3p and negative controls. 

### 4.12. Bioinformatics Analysis of Microarray and Target Prediction

Gene ontology (GO) hierarchy analyses were carried out on the differentially expressed genes using the Gene Ontology Enrichment Analysis Software Toolkit (Version 1.30, Beijing, China). GO was organized into three partially overlapping categories: biological processes, molecular functions, and cellular components. Pathway enrichment analyses of gene expression were obtained using web gestalt WEB-based Gene Set Analysis Toolkit software, which involved databases from the Kyoto Encyclopedia of Genes and Genomes (KEGG). A *p* value reflecting the importance of GO or the pathway results value was used to identify the significant GO terms and pathways. miRWalk, miRanda, miRDB, RNA22, and Targetscan were used for target prediction of miRNAs. For predicting the miRNA-associated mRNA network, we first predicted target mRNAs of abnormally expressed miRNA. Targets identified by more than three prediction tools were selected for further analysis. We then selected common mRNAs with mRNA expression profiles from the predicted mRNAs to visualize a miRNA-associated mRNA network using the Cytoscape (Denver, CO, USA).

### 4.13. Western Blot Analysis

Cellular protein was extracted with cold RIPA buffer containing protease inhibitors (Beyotime, Shanghai, China). Lysates were cleared by centrifugation at 14,000 rpm at 4 °C for 15 min. Protein concentrations were determined using the BCA assay (Thermo Scientific). Aliquots of protein (20 μg) were separated by 10% SDS-PAGE, and the separated proteins were transferred to PVDF membrane. Membranes were blocked with 5% (w/v) non-fat milk in Tris-HCl buffered saline (pH 7.4) with Tween-20 and incubated with the primary, monoclonal antibody overnight at 4 °C. Subsequently, membranes were washed with Tris-HCl buffered saline and incubated with secondary antibodies conjugated to horseradish peroxidase, diluted to 1:3000 (CST) and 1:5000 (Abcam), at room temperature for 1 h. Membranes were washed in Tris-HCl buffered saline, and bounds were detected with SuperSignal West Femto/Pico Kit (Thermo Scientific). Blots were visualized and quantified using a Tanon-5200 Imaging System (Tanon, Shanghai, China).

### 4.14. Luciferase Reporter Assay

Plasmids containing the mutant and non-mutant sequence of 3′UTR of TRAF4 were structured. A Luciferase reporter gene assay was performed using the Dual-Luciferase Reporter assay system (Ribobio) according to the manufacturer’s instructions. Cells of 90% confluence were seeded in 96-well plates with a concentration of 1 × 10^4^/well and incubated for 24 h. Cells were co-transfected with miR-21-3p or negative control and a Luciferase Reporter plasmid. A total of 100 ng of Luciferase Reporter plasmid was mixed with 1.5 p mol of miRNA mimic or negative control in 10 μL of opti-MEM. A total of 0.25 μL of lipofectamine2000 was diluted into 10 μL of opti-MEM and added into the former mixture after incubation for 5 min. When incubated for another 20 min, 20 μL of the transfection mixture and 80 μL of antibiotic-free RPMI1640 media were added into the 96-well plate and incubated at 37 °C and 5% CO_2_. Reporter gene assays were performed 48 h post-transfection using the Dual-Luciferase assay system (Promega, Madison, WI, USA). Firefly luciferase activity was normalized for transfection efficiency using the corresponding Renilla luciferase activity. All experiments were performed at least three times.

### 4.15. RT-QPCR

All primers (Bulge-Loop™ miRNA RT-qPCR Primer kits) for miRNA were purchased from Guangzhou RiboBio Co., Ltd. (Guangzhou, China) All primers for mRNA were synthetized from the GenScript Corporation. Detailed sequences are shown in Table S4.

Total RNA (~2 μg) was extracted using Trizol regent (Invitrogen). cDNA was synthesized using Moloney Murine Leukemia Virus (MMLV) reverse transcriptase (Promega) and ribonuclease inhibitor (Fementas, Madison, WI, USA). SYBR Green mastermix was purchased from Toyobo Technologies (Osaka, Japan). QPCR reactions were run using the StepOnePlus system (Applied Biosystems, Carlsbad, CA, USA). The data for miRNA and mRNA were normalized to U6 and β-actin, respectively. The expressions of miRNA and mRNA were presented as relative RNA expression using ΔΔCq formula (the fold change in target gene expression was equal to 2^−ΔΔCq^). All results were presented as the mean of triplicates ± SD from three independent experiments.

### 4.16. Tumor Xenograft in Nude Mice

Female NOD/SCID mice (6 per group) were subcutaneously injected with 5 × 10^3^ of cells of positive (CD71^−^/CD271^+^/CD338^+^) and negative (CD71^+^/CD271^−^/CD338^−^) cells into the upper limb. The xenografts were monitor for 8 weeks, then the mice were sacrificed by cervical dislocation. Subcutaneous implanted tumors were collected and stained with HE and AE1/AE3 antibody. All animal experiments were conducted in accordance with the protocols approved by the Laboratory Animal Centre of Southeast University (20110226006, 26 February 2011).

### 4.17. Statistical Analysis

Statistical analysis was performed using SPSS 17.0. (Armonk, NY, USA). A *p* value < 0.05 was considered to be statistically significant. Wherever stated, one asterisk denotes *p* < 0.05, two asterisks denote *p* < 0.01, three asterisks denote *p* < 0.001, and NS denotes *p* > 0.05.

## 5. Conclusions

In conclusion, we found that the combination of CD71, CD271, and CD338 (CD71−/CD271+/CD338+) can well identify and isolate CSCs in ESCC. Through inhibiting post-transcriptional factors of TRAF4, hsa-miR-21-3p promoted proliferation and anti-apoptosis, and hsa-miR-21-3p has potential to be a biomarker for the early diagnosis of ESCC. 

## Figures and Tables

**Table 1:** Relative expression of miR-21-3p in esophageal cancer tissues and matched para-cancer tissues.

Group	*n*	Mean ± SD	ΔΔC_T_ *	2^−ΔΔCT^	*p* Value	*t* Value
Tumor tissue	137	10.201 ± 2.930	−0.896 ± 3.865	1.863	<0.01	2.737
Para-tumor tissue	173	11.097 ± 2.809				

* ΔC_T_ = C_T Target gene_ − C_T Reference gene_; C_T_ = (ΔC_T Cancer tissue_ − ΔC_T Para-cancer tissue_).

**Table 2:** Conditional logistic regression analysis of the risk for esophageal cancer of miR-21-3p.

Group	β	SE	*χ* ^2^	*p* Value	OR	95% CI
Tumor tissue	−0.126	0.049	6.644	0.010	1.135	1.030–1.250
Para-tumor tissue					1.000	

SE: standard error; OR: odds ratio; CI: confidence interval.

## Supplementary Materials

The following are available online at https://www.mdpi.com/2072-6694/11/4/518/s1, Table S1: Differently expressed miRNAs in CD71−/CD271+/CD338+ cells detected by miRNA microarrays, Table S2: Diffrential regulation of mRNAs in CD71−/CD271+/CD338+ cells detected by mRNA microarrays (Partial results), Table S3: Differential expression of mRNAs in miR-21-3p mimics transferred and negative control cells by microarray, Table S4: Sequences for PCR primers.

## Abbreviations

CSCs	Cancer stem cells
ESCC	Esophageal squamous cell carcinoma
EdU	5-ethynyl-2′-deoxyuridine
ALDH	Aldehyde dehydrogenase
MTT	Thiazolyl blue tetrazolium bromide
HE	Hematoxylin-eosin
SCID	Server Combined Immune-deficiency
p75NTR	p75 neurotmphin receptor
MMLV	Moloney Murine Leukemia Virus
GO	Gene ontology
KEGG	Kyoto Encyclopedia of Genes and Genomes
ANOVA	Analysis of variance
SSM	Serum-Supplied-DMEM-F12 Medium with 10% fetal bovine serum
SFM	Serum-Free-DMEM-F12 Medium
FACS	Fluorescence activated cell sorting
