# Supplementary material for: Identification of Cancer Stem Cell Molecular Markers and Effects of hsa-miR-21-3p on Stemness in Esophageal Squamous Cell Carcinoma
Source: Cancers (Basel). 2019 Apr 11;11(4):518. doi: 10.3390/cancers11040518 (PMC6521292; doi:10.3390/cancers11040518)
Supplement: Supplementary file 1 [file cancers-11-00518-s001.pdf]

Supplementary Materials: Identification of Cancer Stem Cell Molecular Markers and Effects of hsa-miR-21-3p on Stemness in Esophageal Squamous Cell Carcinoma

Zhikui Gao, Hui Liu, Yajuan Shi, Lihong Yin, Yong Zhu and Ran Liu

Table S1. Differently expressed miRNAs in CD71-/CD271+/CD338+ cells detected by miRNA microarrays.

Systematic Name	Fold change	Regulation	Chromosomal	Mirbase Accession NO.
hsa-miR-1207-5p	1.982873	up	chr8	MIMAT0005871
hsa-miR-638	1.947877	up	chr19	MIMAT0003308
hsa-miR-762	1.9363505	up	chr16	MIMAT0010313
hsa-miR-193a-3p	1.9259949	up	chr17	MIMAT0000459
hsa-miR-3665	1.9120283	up	chr13	MIMAT0018087
hsa-miR-1202	1.9076614	up	chr6	MIMAT0005865
hsa-miR-1915-3p	1.8432146	up	chr10	MIMAT0007892
hsa-miR-197-3p	1.8268632	up	chr1	MIMAT0000227
hsa-miR-940	1.826798	up	chr16	MIMAT0004983
hsa-miR-4281	1.8177478	up	chr5	MIMAT0016907
hsa-miR-3195	1.7528486	up	chr20	MIMAT0015079
hsa-miR-21-3p	1.7219718	up	chr17	MIMAT0004494
hsa-miR-3162-5p	1.6782901	up	chr11	MIMAT0015036
hsa-miR-224-5p	1.6726134	up	chrX	MIMAT0000281
hsa-miR-3180-5p	1.668098	up	chr16	MIMAT0015057
hsa-miR-181d-5p	1.6528	up	chr19	MIMAT0002821
hsa-miR-3196	1.6409832	up	chr20	MIMAT0015080
hsa-miR-29c-3p	1.6334755	up	chr1	MIMAT0000681
hsa-miR-3679-5p	1.632122	up	chr2	MIMAT0018104
hsa-miR-574-3p	1.6287357	up	chr4	MIMAT0003239
hsa-miR-221-3p	1.6075988	up	chrX	MIMAT0000278
hsa-miR-4286	1.6026103	up	chr8	MIMAT0016916
hsa-miR-101-3p	1.5595156	up	chr1	MIMAT0000099
hsa-miR-3125	1.551821	up	chr2	MIMAT0014988
hsa-miR-1246	1.5494092	up	chr2	MIMAT0005898
hsa-miR-18b-5p	1.5464137	up	chrX	MIMAT0001412
hsa-miR-423-5p	1.5381923	up	chr17	MIMAT0004748
hsa-miR-19a-3p	1.5357118	up	chr13	MIMAT0000073
hsa-miR-20a-3p	1.5292501	up	chr13	MIMAT0004493
hsa-miR-18a-5p	1.5276583	up	chr13	MIMAT0000072
hsa-miR-29b-3p	1.5275594	up	chr1	MIMAT0000100
hsa-miR-331-3p	1.5234461	up	chr12	MIMAT0000760
hsa-miR-34b-5p	1.5232713	up	chr11	MIMAT0000685
hsa-miR-148a-3p	1.5231193	up	chr7	MIMAT0000243
hsa-miR-3198	1.5172197	up	chr22	MIMAT0015083
hsa-miR-193b-3p	1.515177	up	chr16	MIMAT0002819
hsa-miR-132	1.5140287	up	chr17	MIMAT0000426
hsa-miR-99b-5p	1.5094832	up	chr19	MIMAT0000689
hsa-let-7b-5p	1.5001699	up	chr22	MIMAT0000063
hsa-miR-1207-5p	1.982873	up	chr8	MIMAT0005871
hsa-miR-638	1.947877	up	chr19	MIMAT0003308
hsa-miR-762	1.9363505	up	chr16	MIMAT0010313
hsa-miR-193a-3p	1.9259949	up	chr17	MIMAT0000459
hsa-miR-3665	1.9120283	up	chr13	MIMAT0018087
hsa-miR-1202	1.9076614	up	chr6	MIMAT0005865
hsa-miR-1915-3p	1.8432146	up	chr10	MIMAT0007892
hsa-miR-197-3p	1.8268632	up	chr1	MIMAT0000227

hsa-miR-940	1.826798	up	chr16	MIMAT0004983
hsa-miR-4281	1.8177478	up	chr5	MIMAT0016907
hsa-miR-3195	1.7528486	up	chr20	MIMAT0015079
hsa-miR-21-3p	1.7219718	up	chr17	MIMAT0004494
hsa-miR-3162-5p	1.6782901	up	chr11	MIMAT0015036
hsa-miR-224-5p	1.6726134	up	chrX	MIMAT0000281
hsa-miR-3180-5p	1.668098	up	chr16	MIMAT0015057

Table S2. Diffrential regulation of mRNAs in CD71-/CD271+/CD338+ cells detected by mRNA microarrays (Partial results).

Transcript ID	Ratio	Chromosomal	Regulation	Symbol
BC015149	7.919	chr3	up	MAP4
BC102036	3.1523	chr3	up	CAV3
NM_021049	2.8632	chrX	up	MAGEA5
BC063835	2.3487	chr7	up	VGF
XM_372302	2.3222	chr10	up	tAKR
BC007023	2.3201	chr11	up	ATM
NM_001008727	2.31	chr19	up	ZNF121
XM_930191	2.2924	chr1	up	LOC647165
BC069585	2.2111	chr12	up	KRTHB6
BC013013	2.1683	chr19	up	ZNF526
BC104846	2.1328	chr21	up	KRTAP20-1
NM_175065	2.1026	chr1	up	HIST2H2AB
NM_004430	1.9968	chr8	up	EGR3
BC047412	1.9938	chr19	up	ZNF433
AY367050	1.8224	chr8	up	RAB11FIP1
NM_000421	1.8196	chr17	up	KRT10
NM_012403	1.8126	chr4	up	ANP32C
AK095562	1.8123	chr19	up	ZNF578
AB208789	1.8088	chr1	up	SCP2
NM_005557	1.7937	chr17	up	KRT16
AF275684	1.7903	chr6	up	PPP1R2P1
XM_927827	1.7783	chr11	up	LOC390211
XM_929465	1.7769	chr6	up	LOC646536
NM_014898	1.7687	chr19	up	ZFP30
BC063697	1.7681	chr12	up	KRT1
X59796	1.6005	chr16	up	CDH5
BC035665	1.5771	chr1	up	HSPA6
NM_001039348	1.5631	chr2	up	EFEMP1
NM_004827	1.5553	chr4	up	ABCG2
AY330488	1.5319	chr5	up	AMACR
BC010958	1.5255	chr12	up	CCND2
NM_021148	1.5247	chr7	up	ZNF273
NM_002275	1.5023	chr17	up	KRT15
NM_002783	0.6654	chr19	down	PSG7
BC093027	0.662	chr6	down	PKIB
NM_031246	0.6588	chr19	down	PSG2
NM_015327	0.6579	chr1	down	SMG5
NM_021629	0.6569	chr3	down	GNB4
BC005807	0.6535	chr10	down	SCD
AK075079	0.6531	chr19	down	PSG1
BC013304	0.6521	chr1	down	SESN2
NM_000516	0.648	chr20	down	GNAS
AY927772	0.6479	chr17	down	BIRC5
BC066921	0.6296	chr3	down	PPP1R2
NM_032336	0.6284	chr8	down	SLD5
XM_931390	0.6185	chr3	down	SETD5

NM_144665	0.6067	chr11	down	SESN3
XM_935180	0.6055	chr2	down	PXDN
NM_018196	0.5663	chrX	down	TMLHE
BC027175	0.5583	chr6	down	HLA-DMB
BC001601	0.5534	chr12	down	GAPDH
NM_003417	0.5471	chr19	down	ZNF264
XM_935183	0.5342	chr2	down	PXDN
BC008600	0.5218	chr17	down	CCL5
BC027454	0.4824	chr8	down	SLD5
NM_019111	0.3996	chr6	down	HLA-DRA
NM_022555	0.3876	chr6	down	HLA-DRB3
NM_000367	0.2605	chr6	down	TPMT

Table S3. Differential expression of mRNAs in miR-21-3p mimics transferred and negative control cells by microarray.

Symbol	Definition	Fold Change	Regulation	*p*-Value
LOC646938	Predicted:similar to TBC1 domain family member 2	14.55	down	1.14E-03
LOC441896	Predicted:partial miscRNA	14.34	down	1.96E-03
NPAS1	Neuronal PAS domain protein 1	11.88	down	5.05E-03
AKR1B10	Aldo-keto reductase family 1,member B10 (aldose reductase)	10.45	down	3.33E-03
TRAF4	TNF receptor-associated factor 4	10.14	down	1.09E-03
SNORD114-2	Small nucleolar RNA，C/D box 114-2	8.93	down	2.28E-02
RBM24	RNA binding motif protein 24	8.76	down	1.41E-02
OR13C9	Olfactory receptor,family 13, subfamily C,member 9	8.55	down	6.84E-03
LOC100133111	Predicted：hypothetical protein LOC100133111	8.17	down	4.79E-02
HS.564000	cDNA clone UI-H-DF0-bep-h-20-0-UI 3	8.14	down	3.47E-02
CYB5R2	Cytochrome b5 reductase 2	15.26	up	2.24E-03
HIC1	Hypermethylated in cancer 1(HIC1),transcript variant 2	13.04	up	2.14E-04
LOC651353	Predicted:similar to cystatin SC	12.79	up	1.87E-03
EGR1	Early growth response 1 (EGR1)	9.28	up	4.09E-04
FDPSL2A	MGC44478 (FDPSL2A), non-coding RNA	9.16	up	2.54E-02
HS.31007	EST176292 Colon carcinoma (Caco-2) cell line II cDNA 5 end	8.98	up	9.98E-03
UNQ9433	RPLK9433	8.77	up	9.08E-03
LOC653497	Predicted:similar to CMT1A duplicated region transcript 4	7.77	up	3.85E-02
PRUNE2	Prune homolog 2 (Drosophila),transcript variant 2	7.25	up	4.96E-02
TMEM217	Transmembrane protein 217	7.06	up	3.05E-02

Table S4. Sequences for PCR primers.

Gene	Orientation	Sequence
Bmi-1	Forward	5′-GGAGACCAGCAAGTATTGTCCTTTTG-3'
	Reverse	5′-CATTGCTGCTGGGCATCGTAAG-3′
p63	Forward	5′-CAGACTTGCCAAATCATCC-3′
	Reverse	5′-CAGCATTGTCAGTTTCTTAGC-3′
involucrin	Forward	5′-TCCTCCTCCAGTCAATACCC-3′
	Reverse	5′-GCTGATCCCTTTGTGTT-3′
cytokeratin 13	Forward	5′-CCATGAAGAGGTGAGCGGGGATTG-3′
	Reverse	5′-CTGTGGGGATGGGAAAGGAAGATGTG-3′
TRAF4	Forward	5′-AGGAG TTCGTCTTTGACACCATC-3′
	Reverse	5′-CTTTGAATGGGCAGAGCACC-3′
β-actin	Forward	5′-CAACTTGATGTATGAAGGCTTTGGT-3′
	Reverse	5′-ACTTTTATTGGTCTCAAGTCAGTGTA CAG-3′
ALDH1	Forward	5′-TGTTAGCTGATGCCGACTTG-3′
	Reverse	5′-TTCTTAGCCCGCTCAACACT-3′
Nanog	Forward	5′-GAACTCTCCAACATCCTGAACCT-3′
	Reverse	5′-TCTGCGTCACACCATTGCTAT-3′
Oct4	Forward	5′-AGAAGGATGTGGTCCGAGTG-3′
	Reverse	5′-GAAGTGAGGGCTCCCATAGC-3′

© 2019 by the authors. Licensee MDPI, Basel, Switzerland. This article is an open access article distributed under the terms and conditions of the Creative Commons Attribution (CC BY) license (http://creativecommons.org/licenses/by/4.0/).
